# A Framework for the Human-Centered Design of Service Processes Enabled by Medical Devices: A Case Study of Wearable Devices for Parkinson’s Disease

**DOI:** 10.3390/ijerph21101367

**Published:** 2024-10-16

**Authors:** Sara Vannelli, Filippo Visintin, Clio Dosi, Laura Fiorini, Erika Rovini, Filippo Cavallo

**Affiliations:** 1 Dipartimento di Ingegneria Industriale, University of Florence, Viale Morgagni 40/44, 50134 Florence, Italy; filippo.visintin@unifi.it (F.V.); laura.fiorini@unifi.it (L.F.); erika.rovini@unifi.it (E.R.); filippo.cavallo@unifi.it (F.C.); 2 Dipartimento di Scienze Aziendali, University of Bologna, Via Capo di Lucca 34, 40126 Bologna, Italy; clio.dosi@unibo.it; 3 The BioRobotics Institute, Scuola Superiore Sant’Anna, Viale Rinaldo Piaggio 34, Pontedera, 56025 Pisa, Italy

**Keywords:** human-centered design, service process innovation, medical device, Parkinson’s disease, technology-enabled service

## Abstract

The successful introduction of medical devices (MDs) in real-world settings hinges on designing service processes that cater to stakeholders’ needs. While human-centered design (HCD) approaches have been widely applied to service process innovation, the literature lacks a methodology that leverages MDs’ key features to design service processes that meet stakeholders’ needs. This study aims to fill this gap by developing a framework for the HCD of service processes enabled by MDs. The proposed framework mixes and adapts methodological elements from HCD and technology-enabled design approaches and proposes four new tools. The five-phase framework was applied to the design of a new Parkinson’s disease diagnosis and treatment process (PD-DTP) enabled by two wearable MDs for the detection of motor symptoms. The case study lasted five months and involved 42 stakeholders in 21 meetings (interviews, focus groups, etc.). Thanks to the case study, the framework was tested, and a new PD-DTP that could benefit all stakeholders involved was identified. This study provides a framework that, in addition to contributing to theory, could assist MDs developers and healthcare managers in designing service processes that cater to stakeholders’ needs by leveraging MDs’ key features.

## 1. Introduction

The introduction of medical devices (MDs) can bring significant improvements to the effectiveness and efficiency of healthcare processes [1,2]. However, to thoroughly predict the benefits MDs’ introduction can bring to stakeholders, a deep understanding of the service processes enabled or supported by the MDs is necessary [3,4]. This lack of understanding could hinder MD designers from effectively communicating the potential advantages of MD adoption and impair healthcare managers’ ability to select MDs that can significantly impact the processes they are responsible for. However, designing a service process around an MD is not a straightforward task, as the introduction of MDs often necessitates substantial changes in the service processes they target (e.g., altering stakeholder roles, introducing activities, activating new resources, etc.) [5]. It involves not only automating certain process steps but also fundamentally transforming how the service is delivered (e.g., deciding how the MD will be deployed, where it will be used, by whom it will be used, on which patients it will be used, etc.). In addition, an MD initially designed for a specific purpose may enable unforeseen improvements within the service process. The continuing rapid advancement of MDs adds another layer of complexity to the design process. As MDs evolve at an unprecedented pace, they often incorporate features that health systems may not be fully prepared to integrate. The design process must assess the ability of healthcare providers to adapt their processes accordingly and anticipate the changes they would need to implement. Therefore, a structured methodology is essential to support the design of service processes enabled by MDs.

Over the years, two main service design approaches have been employed: technology-enabled design (TED) and human-centered design (HCD) approaches. TED approaches aim to enhance service processes by integrating the MD into them (e.g., improving customer experience or increasing operational efficiency) [6,7,8]. They usually leverage tools like service mapping, workflow redesign, and requirements identification [9]. Unfortunately, TED approaches have failed to bridge the gap from MD development to its actual use in real-world settings because they focus on MD functions, failing to address stakeholders’ needs. Indeed, the success of introducing new MDs hinges on implementing service processes that cater to the needs of potential stakeholders [4,10]. This is what HCD approaches do: following an HCD approach, the solution (e.g., the service process, the MD, etc.) is designed to address stakeholders’ needs [11]. Researchers have employed a vast range of HCD approaches to design healthcare service processes, which could differ in the stakeholders included in the design process, the stakeholders’ contribution, the phases of the design process, and the employed tools. Concerning the stakeholders included, distinctions can be made between patient-centered design approaches, where only patient preferences, needs, and values are taken into account [12,13,14], and multi-stakeholder design approaches [10,15,16,17,18], where healthcare professionals and institutional/organizational actors’ perspectives are taken into account, to keep track of the interrelation among professionals [19,20] and the complexity of healthcare services [15]. Regarding the stakeholders’ contribution, distinctions can be made between co-design approaches, where stakeholders are actively involved in all stages of the creative process [21], and more traditional approaches, where design is primarily driven by the design team (i.e., a group of individuals who collaborate to design services [22]) [23]. Concerning the phases of the design process, some widely employed methods are the Double Diamond model, which contains two sequences of diverging and converging [24], and the three-phase HCD IDEO Field Guide to HCD [25]. Finally, several tools could be employed to encourage stakeholder participation and support the design team, such as Personas [26] and Customer Journey Maps [27]. Design thinking is the more widely employed HCD approach that prioritizes stakeholders’ needs by observing how people interact with their environments and employs the iterative co-design of innovative services [28,29]. However, when an MD has already been selected, employing traditional HCD approaches may not be wise. HCD approaches prioritize stakeholders’ needs over MD functions, which are conceptualized as solutions to the identified stakeholders’ needs. However, it is not worthwhile to start by broadly investigating stakeholders’ needs (as traditional HCD approaches do) since the needs the new service process will address are the only ones the selected MD could meet [30,31]. Despite all the studies that have applied HCD approaches to innovate service processes in healthcare [24,32], the literature lacks a methodology to apply HCD approaches to design service processes that address stakeholders’ needs by leveraging a selected MD. To succeed in this effort, we should rely on a methodology focusing on both MD functions and stakeholders’ needs. Even when MDs are designed following HCD approaches [33,34,35], a limited effort is made to design service processes that meet the needs of stakeholders by leveraging the MD. Typically, service design is limited to business modeling [36,37].

This study seeks to address this gap by developing a five-phase framework that adapts phases and tools typical of HCD and TED approaches to the scenario where an MD will necessarily be part of the new service process. Furthermore, the framework presents new tools that have been purposely identified. The framework was tested by applying it to the design of a new Parkinson’s disease diagnosis and treatment process (PD-DTP) enabled by two wearable MDs for motor symptoms detection, namely SensHand and SensFoot [38] (hereafter, “SensH&F”). This setting is particularly relevant and challenging. Parkinson’s disease (PD) is a widespread neurodegenerative disease with huge economic and social costs [39]. The latter is due to the disabling motor (e.g., tremor, postural instability, and muscle stiffness) and nonmotor symptoms (e.g., depression, anxiety, and cognitive impairment) that substantially reduce patients’ quality of life and the massive burden this disease places on patients’ families [40]. Moreover, PD-DTP is complex and long, with patients interacting with many specialists and their conditions varying along the course of the disease [41]. Finally, although wearable MDs for symptoms detection are very promising [42,43,44,45] and SensH&F represent state-of-the-art MDs for PD [43], their use in medical practice is still relegated to pilot cases [44].

This paper proposes a framework for the HCD of service processes enabled by MDs. It fills a critical gap in the literature by integrating TED perspectives into HCD methodologies, but it also provides healthcare managers and MD designers with a flexible framework they could employ in future projects. Moreover, the framework was applied to the case of the design of a new PD-DTP enabled by two innovative wearable MDs. Through this application, this study successfully tested the framework while offering insights for managers and practitioners involved in PD-DTP management and innovation.

## 2. Materials and Methods

This study proposes a five-phase framework that supports the design of a new service process that addresses stakeholders’ needs by leveraging an MD. The framework mixes and adapts existing methodological elements from HCD (e.g., identification of relevant stakeholders, stakeholders’ needs, and current service process’s problems) [46,47] and TED (e.g., identification of MD’s key features) [9] approaches to develop a methodology that focuses on both MD functions and stakeholder needs without prioritizing one element over the other. To achieve this, the framework proposes four new purposely developed tools, such as the Problem–Features–Benefit matrix (see Appendix C for a description of the new tools). Table 1 compares the typical HCD and TED approaches and describes what the proposed framework inherits and adapts from them. The framework was tested on the design of a new PD-DTP enabled by two wearable MDs for motor symptoms detection. This section presents the framework and the case informants.

### 2.1. A Framework for the Design of New Service Processes Enabled by MDs

**Figure 1:**
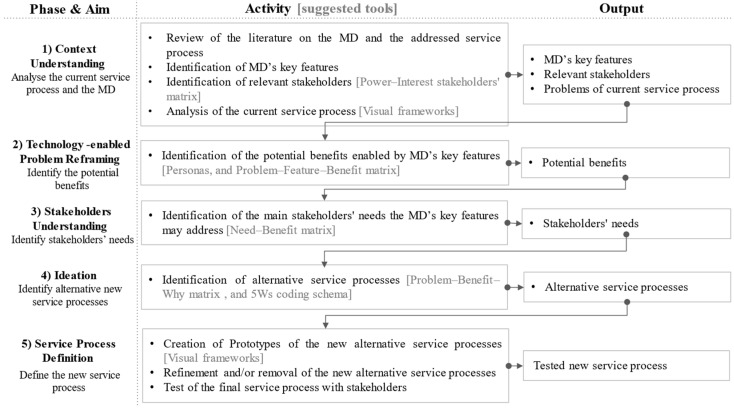
Framework for the HCD of service processes enabled by MDs.

The framework comprises five phases: Context Understanding, Technology-enabled Problem Reframing, Stakeholder Understanding, Ideation, and Service Process Definition. Each phase has one or more aims, one or more activities that must be carried out to achieve them, and one or more outputs that are inherited by the downstream phases (Figure 1). The framework is conceived as a versatile blueprint with tools that can be customized to suit the specific application. Some tools that can be used to carry out the activities are suggested in Figure 1 and in the following description of the phases.

#### 2.1.1. Phase 1: Context Understanding

**Figure 2:**
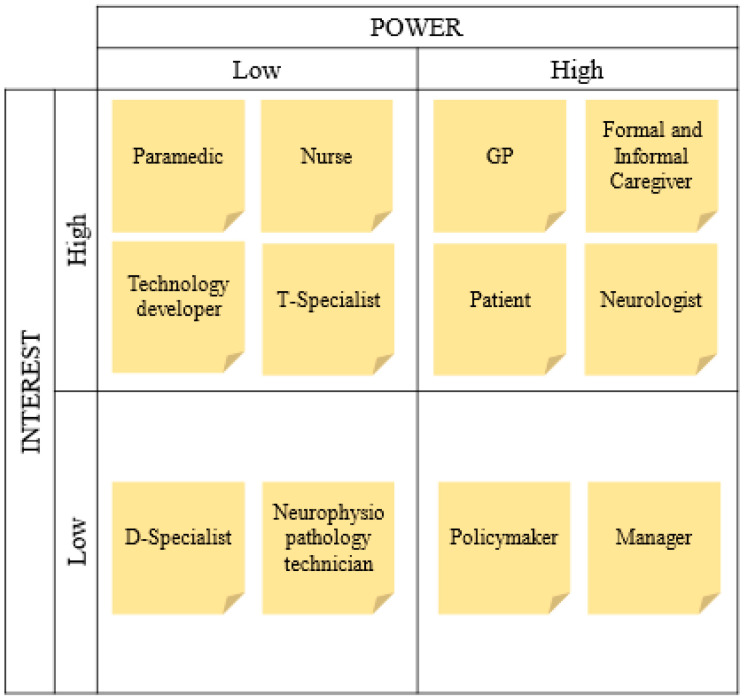
Power–Interest stakeholders’ matrix.

The design process starts with a clear focus on the MD and the service process where the MD could be introduced. The literature on the MD could be reviewed, and meetings with MD providers and/or developers could be organized to identify the MD’s key features (i.e., the features that characterize the MD and make the MD distinguishable from others). Concurrently, the best practices and national and international guidelines on the addressed service process are reviewed to glimpse its main problems and whether and how the MD’s key features could address them. Visual frameworks, such as the Process Chain Network (PCN) diagrams [48,49,50], could be employed to gain insights into the problems of the current service process. After that, relevant stakeholders are identified to select the points of view to include in all the following phases. The Power–Interest stakeholders’ matrix [51,52] could be employed to identify relevant stakeholders (Figure 2). Interest is high if the stakeholder’s job is likely to be significantly impacted by a redesign of the PD-DTP, while power is high if the stakeholder can significantly influence the success of the implementation. Stakeholders are relevant if they have both high interest and high power. This analysis follows the MD’s key features identification and best practices and national and international guidelines review because it requires knowing the stages of the service process that the MD’s key features can reasonably impact (e.g., if the MD’s main features could reasonably be expected to impact only the diagnosis phase of the PD-DTP, we may not include physiotherapists in the following phases as they are not involved in the diagnosis). Finally, the service process’s main problems are refined by discussing with relevant stakeholders (in individual or group interviews, focus groups, etc.).

#### 2.1.2. Phase 2: Technology-Enabled Problem Reframing

This phase links the potential benefits of the MD (i.e., the desired or expected results that could be achieved by introducing the MD) with the main problems of the current service process [31]. The Problem–Feature–Benefit matrix is suggested to convert identified problems within the targeted service process into potential benefits of the new service process. This purposely developed tool is described in Appendix C. The information obtained from the meetings held in Phase 1 will probably be sufficient to do this, but additional meetings with relevant stakeholders could be employed to refine the potential benefits and link them with the problems of the current service process.

#### 2.1.3. Phase 3: Stakeholders Understanding

This phase aims to identify the stakeholders’ needs that the MD addresses. HCD methodologies typically start with identifying stakeholders’ needs [53], but doing this might lead to identifying needs that cannot be met with the chosen MD. Therefore, the framework investigates stakeholders’ needs only at this stage, and this phase focuses only on those needs linked to the potential benefits the MD’s key features enabled. The Need–Benefit matrix is suggested to extract stakeholders’ needs that the identified potential benefits could address. This purposely developed tool is described in Appendix C. Again, the information obtained from the meetings held in the previous phases may be sufficient to pursue this aim, but additional meetings with relevant stakeholders could be employed to identify stakeholders’ needs.

#### 2.1.4. Phase 4: Ideation

Phase 4 seeks to identify alternative service processes enabled by the MD that meet the identified needs. Brainstorming meetings with the relevant stakeholders are held to generate ideas to address the stakeholders’ needs. These ideas may also refer to single steps in the service process. Then, all the ideas are carefully coded and organized to determine how they can be made up into service processes. The Problem–Benefit–Why matrix and the 5Ws coding schema are suggested to codify the ideas. The two purposely developed tools are described in Appendix C.

#### 2.1.5. Phase 5: Service Process Definition

This final phase aims to identify a viable, desirable, and feasible service process [54]. It is an iterative phase that entails testing prototypes with relevant stakeholders. The prototype provokes more accurate feedback from relevant stakeholders, allowing the service process to be refined or discarded in the worst case [25]. Prototypes could be rough artifacts (e.g., a story or a video addressing only partial elements of the service process) since this iterative process should be fast [15,55,56]. Again, visual frameworks could be employed to represent the designed service processes.

**Table 1:** A comparison of TED and HCD approaches and a description of their relationships with the proposed framework.

Approaches	*TED Approaches*	*HCD Approaches*
**Constraints and objectives**	Constraints: the MD and the service process are given at the beginning of the design process. Objectives: enhance the service processes by integrating the MD into them (e.g., improving customer experience).	Constraints: the MD is not given at the beginning of the design process, but the targeted service process might be given. Objectives: design an MD that addresses stakeholders’ needs and/or redefine the service to address the stakeholders’ needs.
**Primary focus**	MD functions.	Stakeholders’ needs.
**Problem emerging when you want to redesign the service given an MD**	Starting by identifying the functions and requirements of the MD without considering stakeholders’ needs could forbid the identification of not-easily-foreseeable uses of the MD and could limit the acceptability and desirability of the solutions for stakeholders.	They typically start with identifying stakeholders’ needs. Doing this might lead to identifying needs that cannot be met with the chosen MD.
**What the proposed five-phase framework inherits**	In Phase 1, the MD’s key features and requirements are investigated, and the current service process is analyzed to identify its main problems.	In Phase 3, there is a deep analysis of stakeholders’ needs (Phase 3). In Phase 4, alternative solutions are brainstormed with stakeholders. In Phase 5, the prototyping and testing of solutions are carried out with stakeholders.
**What the proposed five-phase framework changes**	Phase 2 (and the related “Problem–Feature–Benefit matrix” described in Appendix C) supports the design team to reframe the (given) potential benefits of the MD in light of the main problems of the current service process. Phase 4 is added to explore multiple ways of including the MD in the service process (see “5Ws coding schema” in Appendix C).	Stakeholders’ needs are investigated only in Phase 3 (and not at the beginning of the design process), and the needs analysis focuses only on needs that are linked to the potential benefits offered by the MD’s key features (see the “Need–Benefit matrix” in Appendix C).

### 2.2. Case Informants

In applying the five-phase framework to the case, 21 meetings with 42 stakeholders were organized (Table 2). Some stakeholders were involved in multiple meetings. All stakeholders had previous experience with PD-DTP. All meetings were held online because of the COVID-19 pandemic. The meetings were recorded, transcribed, and coded.

## 3. Results

This section presents the framework’s application to the chosen case: the design of a new PD-DTP enabled by wearable MDs for motor symptom detection.

### 3.1. Phase 1—Context Understanding

Phase 1 explored SensH&F’s key features, current PD-DTP main problems, and relevant stakeholders. SensH&F’s key features (KFs) were identified by discussing with SensH&F developers and triangulating their responses against the scientific literature [43,45,57,58,59,60]. SensH&F are designed for joint use: patients wear the three sensing elements of SensHand on the wrist, thumb, and index finger of the hand and SensFoot on the dorsum of the foot (both on the left and right side) while performing the motor tasks for upper and lower limbs that are suggested in Section III of the Movement Disorders Society Unified Parkinson’s Disease Rating Scale (MDS-UPDRS) [61]. SensH&F can accurately (KF1 = Accurate detection) and objectively measure (KF2 = Objective detection) the motor performance of the patients during exercises and may detect motor impairments even when they are undetectable by the naked eye or by other MDs (KF3 = Fine-grained detection). Moreover, they are easy to use (KF4 = Easy to use). Indeed, they are easy to wear, the session that the patient has to do while wearing them lasts about 15 min, and the exercises are always the same and are identical to those that patients are accustomed to performing during neurological examinations. Finally, they are easily transportable (KF5 = Easy to transport) because they are lightweight and robust.

This study focused on the PD-DTP adopted in the Tuscany region (Italy) [62]. This PD-DTP was compared against those adopted in other Italian regions (e.g., PD-DTP adopted by the Puglia region [63]), the best practices available in the literature (e.g., the “PRIME-Parkinson” model [64] and the “personalized care management” model [65]), and national (e.g., LIMPE-ISS 2013 [66]) and international guidelines (e.g., MDS-UPDRS scale [61]) for PD-DTP. The analysis revealed that the PD-DTP adopted in the Tuscany region is consistent with all of these and that SensH&F have not been adopted yet in clinical practice.

The Power–Interest stakeholders’ matrix [51,52] was employed to identify the relevant stakeholders (Figure 2). Stakeholders are divided into (i) patients; (ii) formal and informal caregivers; (iii) health service providers, who are subdivided into those focused exclusively on the diagnosis process (referred to as D-specialists, e.g., neuroradiologists, neurogenetics, nuclear medicine doctors, etc.), those focused exclusively on the treatment process (referred to as T-Specialists, e.g., physiotherapists, physiatrists, phoniatricians, etc.), and General Practitioners (GPs), neurologists, nurses, paramedics, and neurophysiopathology technicians; (iv) SensH&F developers; (v) policymakers (e.g., national authorities, policy developers, or advisors); and (vi) healthcare managers (at different levels: hospitals managers, territorial clinics managers, neurology units directors, etc.). Given the high number of stakeholders, the following meetings mainly included stakeholders with both a high level of interest and a high level of power. Other stakeholders were included when necessary. For example, nurses were included to understand the conditions that would incline them to use SensH&F, and managers to obtain insights into the feasibility of using SensH&F in territorial or specialized healthcare facilities).

**Figure A1:**
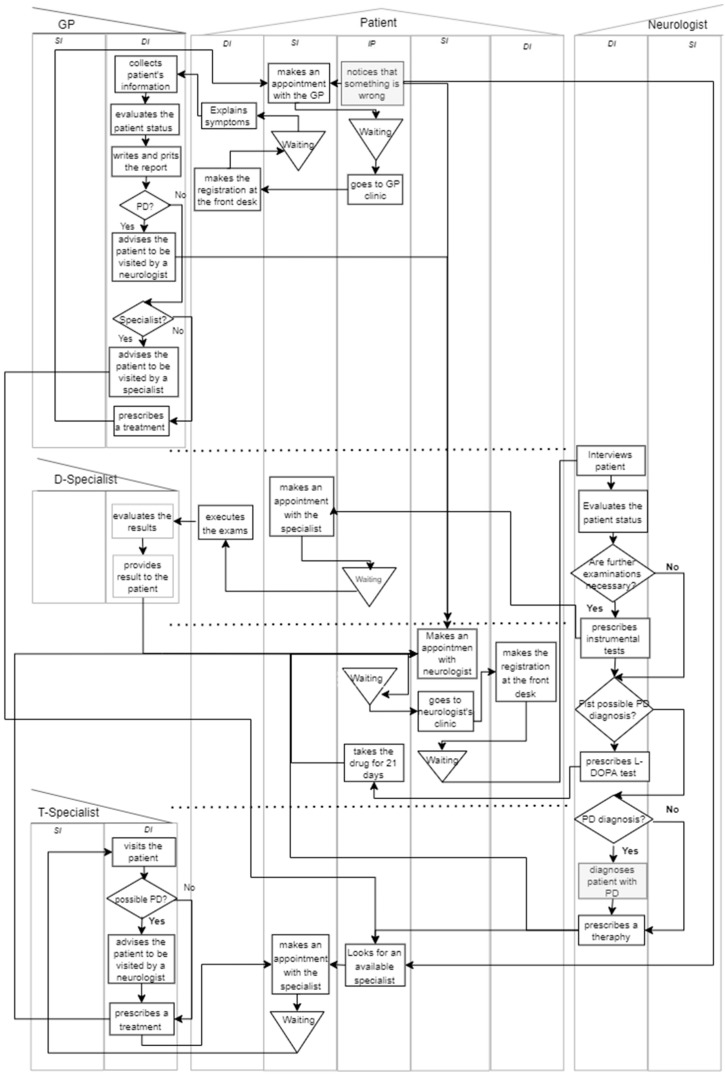
The current PD-DTP—from the appearance of motor symptoms to the neurological diagnosis.

**Figure A2:**
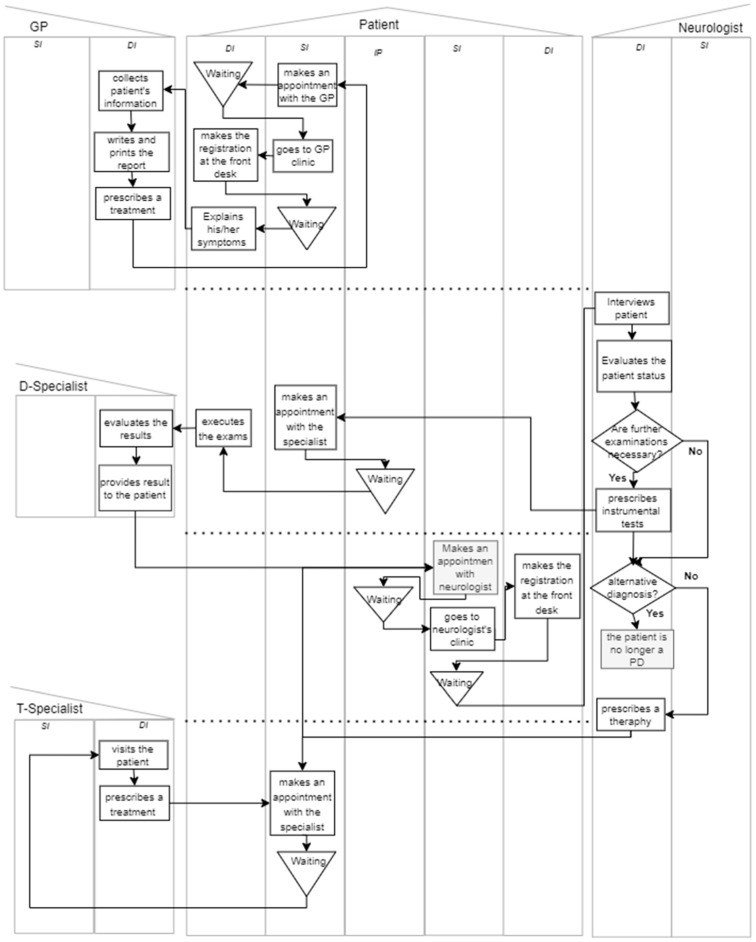
The current PD-DTP—from the neurological diagnosis onward.

Identifying current PD-DTP problems relied on secondary sources [67,68,69] and six semi-structured interviews with seven stakeholders (Table 2). The interviews were all single interviews, except for the one with the PD patient, who was interviewed with his informal caregiver (both PD_1 and ICG_1 participated in INT4; see Table 2). The interviews were structured following the main principles of Service Process Audit [48]. SensH&F’s key features were considered when identifying the main problems. Indeed, we first described SensH&F to the interviewees, and then we asked them to describe the current PD-DTP, whether the process steps were valuable or problematic, and whether SensH&F could address the identified problems. That way, we focused only on problems that SensH&F could reasonably address. For example, we only deal with the problems connected to the detection of motor symptoms since SensH&F only detect those, omitting problems dealing with detecting one of the other symptoms PD patients usually have. Five main PD-DTP problems were identified: Late diagnosis, Subjective assessment, Inaccurate assessment, Infrequent assessment, and Improper timing. The Late diagnosis problem (P1) arises because the diagnostic process is usually triggered by the onset of motor symptoms that only emerge when the neuronal population is largely compromised. Moreover, these symptoms are often misinterpreted or ignored. This prompts patients, often following the GP’s advice, to delay diagnosis by turning to other specialists, such as the orthopedist. The Subjective assessment (P2) and the Inaccurate assessment (P3) problems stem from using qualitative scales (e.g., mild, severe, or continuous tremor) to evaluate the patient’s motor performance while executing the standard exercises in Section III of the MDS-UPDRS scale [61]. These scales make it difficult to track deteriorating motor performance reliably, especially when the patient is seen infrequently and by different neurologists (as is often the case). The Infrequent assessment problem (P4) is related to the limited capacity of health systems compared to the number of PD patients needing care. Infrequent re-evaluations can sometimes result in pharmacological therapies not being administered at all: “*The L-DOPA diagnostic test sometimes is not prescribed due to the impossibility of the neurologist to re-evaluate the patient’s condition frequently*” (NEURO_3). Finally, the Improper timing (P5) follows from the fact that the motor assessment is based on the performance observed by neurologists during visits. Indeed, especially if patients suffer from motor fluctuations (i.e., the transition between periods of good and poor symptom control), the timing of the visit may not be the most appropriate for assessing the actual patient’s conditions. Usually, neurologists ask patients to keep notes of their motor conditions over time in a diary, but, especially in subjects with declining cognitive performance, significant recalling bias and inaccuracy typically affect patients’ self-assessments. P2, P3, P4, and P5 negatively impact the therapy prescription because they prevent a proper evaluation of therapeutic effects. After the interviews, PCN diagrams [48,49,50] were employed to gain insights into current PD-DTP problems. Through PCN diagrams, the as-is PD-DTP from the appearance of motor symptoms to the neurological diagnosis (Figure A1 in Appendix A) and the neurological diagnosis onward (Figure A2 in Appendix A) were represented. In all PCN diagrams, the first and last process steps are colored gray.

### 3.2. Phase 2—Technology-Enabled Problem Reframing

Phase 2 aimed to identify the potential benefits that SensH&F’s key features enabled. Two focus groups with three neurologists each (Table 2) were conducted to glimpse SensH&F’s potential benefits [60]. Then, considering the literature on the MDs and the insights obtained from the interviews held in Phase 1, the identified problems were converted into potential benefits using the Problem–Features–Benefit matrix (Table 3). This purposely developed tool identifies how (i.e., thanks to which key features) the MD could address a certain problem. SensH&F’s key features may address the identified problems. First, by providing an accurate (KF1) and objective (KF2) evaluation of motor performances, they may facilitate a more objective (B2 = Objective assessment) and accurate (B3 = Accurate assessment) assessment of motor symptoms. B2 may help avoid subjective assessment of patients’ status (P2), while B3 may help avoid its inaccurate assessment (P1). Second, they may facilitate the early detection of symptoms (B1 = Early detection) by detecting even subtle motor symptoms that cannot be observed by the naked eye (KF3). B1 may help diagnose PD earlier (P1). Third, their easy-to-use nature (KF4) may make it possible to delegate [70] the execution of the exercises to people other than the neurologist (e.g., nurse, GP, formal and informal caregiver) and/or to the patient themselves (B4 = Delegate). Finally, SensH&F’s transportable nature (KF5) may allow the motor assessment to be performed in the patient’s home or at conveniently located clinics (B5 = Change setting). B4 and B5 may help improve the frequency of evaluations of the patient’s condition (P4), even when traditional health services are inactive (P5).

### 3.3. Phase 3—Stakeholders Understanding

**Figure A3:**
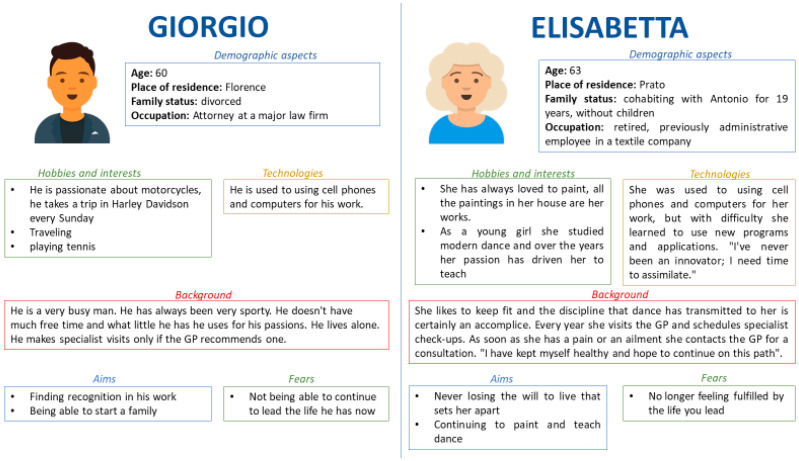
Personas in the prodromal PD (Giorgio) and the early PD (Elisabetta).

**Figure A4:**
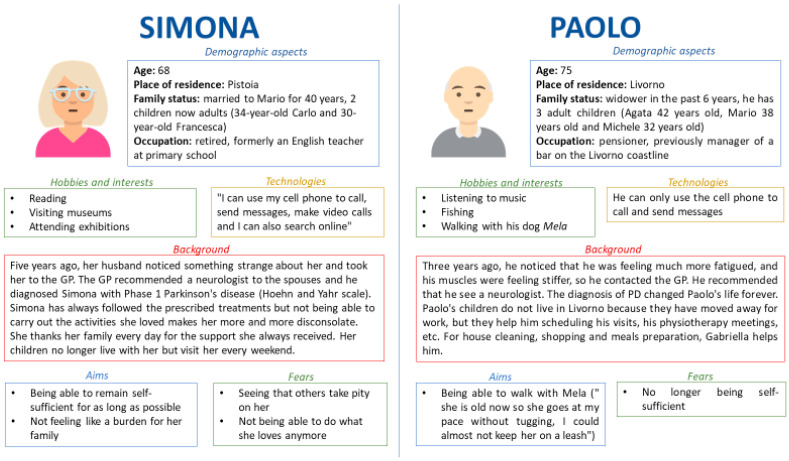
Personas corresponding to PD patients.

This phase aimed to identify the main stakeholders’ needs (Ns), focusing only on the ones addressed by the potential benefits enabled by SensH&F’s key features. For example, one patient might need to “Feel safe while walking”, but SensH&F’s potential benefits do not address this need, so designing solutions around that need is out of the project’s scope. Given the wealth of information obtained from the interviews held in Phase 1, no additional meetings were organized to identify stakeholder needs. From the transcripts of the six interviews conducted in Phase 1, the stakeholders’ needs were extracted using the Need–Benefit matrix (Table 4). This purposely developed tool identifies stakeholders’ needs that SensH&F’s potential benefits could address. Patients are the most heterogeneous stakeholder group, so different patients might have different needs. Therefore, Personas—i.e., detailed fictional representations of the typical service users [71]—were employed to better understand their needs and facilitate discussion [26]. Specifically, four personas were identified: Giorgio (Figure A3 in Appendix B), Elisabetta (Figure A3 in Appendix B), Simona (Figure A4 in Appendix B), and Paolo (Figure A4 in Appendix B). Giorgio represents patients in the prodromal stage of PD (the stage in which PD has already begun its course, but there are also no apparent symptoms), unaware of their status. Elisabetta represents patients in an early stage of the disease (patients with initial mild motor symptoms), unaware of their status. While Elisabetta performs periodic health checks, Giorgio does not. Thus, Giorgio would need the National Health System to push him for check-ups so that he could receive the diagnosis as soon as possible (N1). The wrong specialists have visited Elisabetta; thus, she needs better coordination between the specialists she consults to be referred to the neurologist sooner (N2). Early detection of motor symptoms through SensH&F (B1) may help in this respect. Simona and Paolo represent patients who have already been diagnosed with PD. Simona is still self-sufficient and has the support of her husband and children. She does not fear technology and regularly uses her smartphone to access apps and the web. Her greatest concern is becoming a burden for her family members. Paolo, by contrast, is not self-sufficient anymore; he needs the constant support of a formal caregiver. He uses the telephone to interact with his children, who live far away, as he is not accustomed to nor able to interact with other devices. Paolo relies on the support of his caregiver to interact with doctors and book visits, which has reduced his frequency of being re-evaluated and having his therapies updated. The objective assessment of motor symptoms (B2) may facilitate the collaboration between doctors and caregivers (N5) and then help informal caregivers in managing PD (N4). Having an accurate assessment of motor symptoms (B3), coupled with the possibility of delegating the assessment (B4) and performing it in additional settings (B5), may help doctors in assigning the proper therapies (N6) and consequently improving the patient’s QoL (N3). Moving to GPs, enabling an early detection (B1) answers the GPs’ need to activate a proactive diagnosis process (N7), allowing them to refer patients to the correct specialists as soon as possible. Moreover, an objective and accurate assessment (B2 and B3) helps GPs to interact with the specialists to take care of the patient over time. B4 and B5 may answer GPs’ need to monitor patients’ health status throughout the disease. In other words, correctly grasping the identified potential benefits can enable GPs to play the role of “care coordinator” [50] effectively (N8)—a burden that has, to date, mostly wrongly fallen on patients [72,73]. Moving to the neurologists, an early detection (B1) responds to the neurologist’s need to administer therapy as soon as possible to postpone the onset of severe symptoms (N9). In addition, relying on an objective (B2) and accurate (B3) assessment, as well as the possibility of delegating the assessment (B4) to other people or of doing it in other settings (B5), may reduce the risk of making errors in the diagnosis and/or treatment process (N10). Finally, relying on an objective (B2) and accurate (B3) assessment may be essential in those settings (e.g., hospital Parkinson’s units) where, due to capacity constraints, patients are not necessarily visited by the same neurologist over time (N11). In these cases, neurologists need to assess the evolution of the disease based on the patient’s description of the symptoms and/or the qualitative reports of neurologists who have seen the patient in the past. However, different neurologists may apply different interpretations to both the patient’s motor performance and the scale used to measure it.

### 3.4. Phase 4—Ideation

Four brainstorming meetings with stakeholders were organized to identify alternative service processes enabled by the introduction of SensH&F that address stakeholders’ needs. The meetings aimed to analyze what could be changed in the current PD-DTP to satisfy the previously defined stakeholder needs. The meetings started with a video showing how to wear SensH&F and perform the relevant exercises; in this way, all stakeholders could envision SensH&F’s usage in their contexts. Then, the brainstorming moderator presented the stakeholders’ needs and asked participants trigger questions. Each participant had to write down at least three ideas on cards and then share them with the group to start a discussion. For example, here are two of the trigger questions that were asked concerning the four patients’ personas:

- “How could we diagnose Giorgio with the disease?” (N1)
- “How could we assess Simona’s status accurately and continuously?” (N3, N4)

Then, stakeholders were asked to work on “analogies” [74,75], i.e., to compare the situation at hand with similar situations/problems they had personally experienced. These are some of the questions asked:

- “Please, think of other MDs employed in supporting the diagnosis of complex diseases. How might we take inspiration from them to achieve an early diagnosis of PD?” (B1)
- “Please, think of other contexts in which the patients’ status assessment is achieved by performing individual, pre-defined exercises that are the same for each session; can we take inspiration from them?” (B4)

Brainstorming meetings were organized until no more interesting ideas emerged. After the meetings, the received ideas were codified using the Problem–Benefit–Why matrix (Table 5). The matrix specifies the aim (“*Why*” column) the MD could have in addressing the identified problems, thanks to the potential benefits it offers. Contextualized to the case, the Why column shows the aims the SensH&F test could have in the new PD-DTP to address the identified needs. The SensH&F test could address the problem of having a late diagnosis (P1) by helping to detect PD in patients in the prodromal stage (WHY1 = Prodromal diagnosis) or in patients already presenting suspicious motor symptoms (WHY2 = Early diagnosis). The SensH&F test could address the problem of having a subjective (P2) and inaccurate (P3) diagnosis by informing periodic neurological visits (WHY3 = Periodic assessment). Finally, the SensH&F test could address the problem of infrequent assessment (P4), usually performed with improper timing (P5), by helping in monitoring patients’ status between neurological visits (WHY4 = Monitoring).

**Figure 3:**
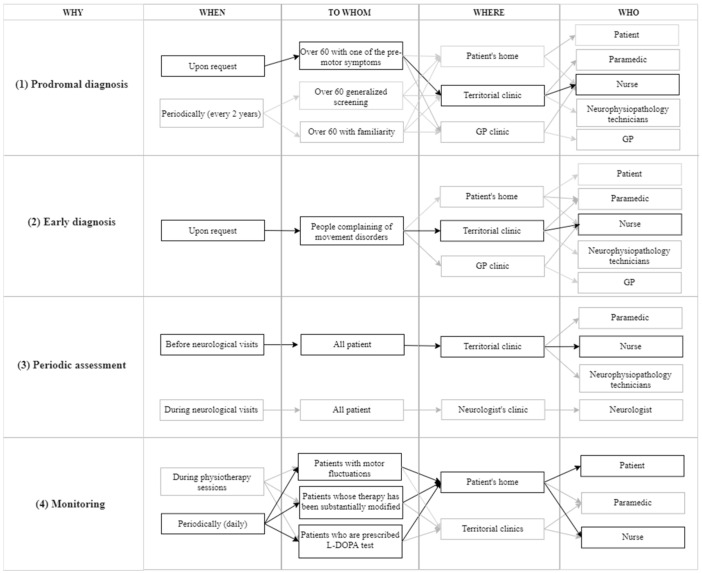
Possible roles of SensH&F test in new PD-DTP following 5Ws coding schema.

Then, starting from the Problem–Benefit–Why matrix, the received ideas were synthesized following the 5Ws coding schema (Figure 3). The purposely developed coding system classified the ideas based on five codes: Why (what could be the aim of the SensH&F test in the new PD-DTP?—it comes from the Problem–Benefit–Why matrix), When (when and how often may the SensH&F test be performed?), to Whom (who may be the target population?), Where (in which setting may SensH&F test be performed?), and Who (who may administer SensH&F tests?). Each idea corresponds to a block. Each “path” linking the different block types represents an alternative configuration for pursuing the identified aim (see Figure 3). During coding, ideas and paths were excluded (gray blocks and arrows in Figure 3) when refuted by the literature and/or the evidence collected in the previous meeting (Table 6 and Table 7 provide insight into the process that led to excluding or retaining ideas). For example, all meeting participants strictly discarded the idea of GPs going to patients’ homes to perform SensH&F tests as this could not be sustainable for GPs.

### 3.5. Phase 5—Service Process Definition

Phase 5 ends with selecting one desirable, technically feasible, and economically sustainable service process. A new PD-DTP corresponds to a combination of one or more paths (one or more aims the SensH&F could have in the service process). Ad hoc prototypes (storyboards of the Whys in which the SensH&F could be employed in the PD-DTP) were developed to collect feedback on all the identified ideas and paths of ideas (Figure 3). Six co-design meetings were conducted. Opinions were sought until convergence was reached. In the end, the paths of ideas (roles of the MD) that were not discarded were assembled to form a new PD-DTP that was desirable, feasible, and viable (see Appendix A).

**Figure A6:**
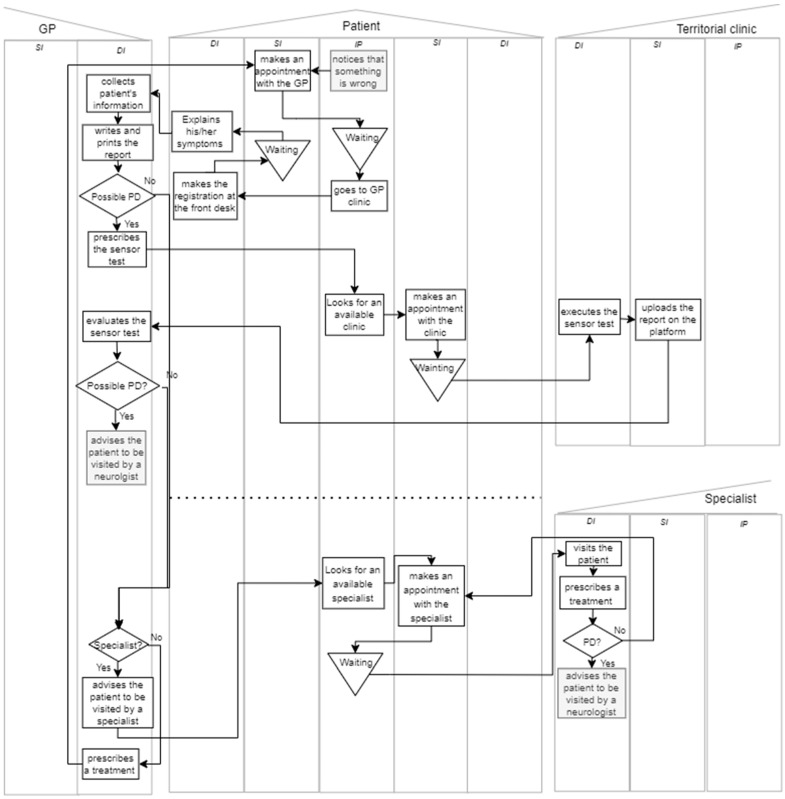
The new PD-DTP—diagnosis triggered by motor symptoms.

**Figure A7:**
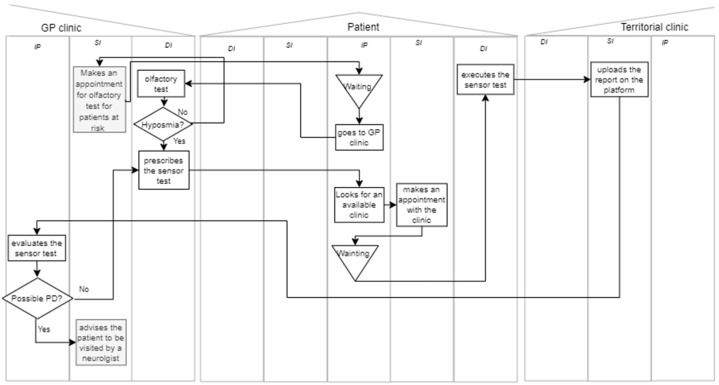
The new PD-DTP—prodromal diagnosis.

**Figure A9:**
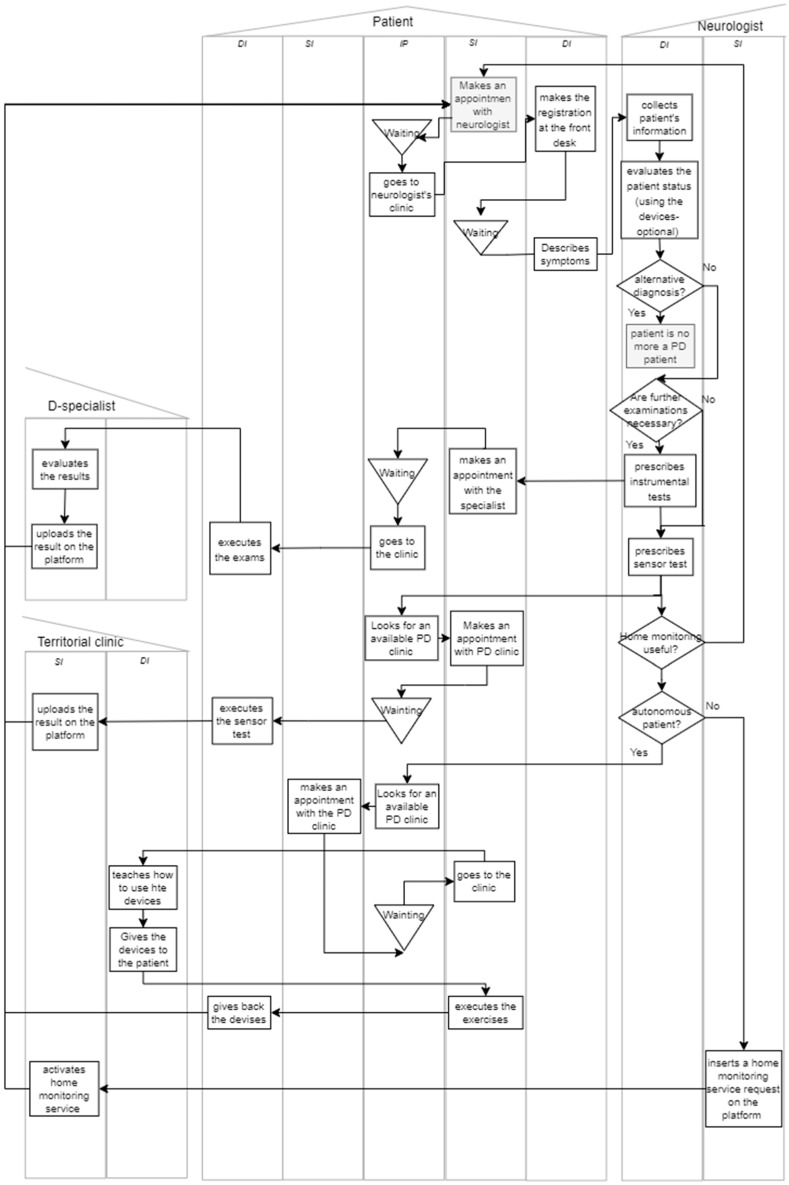
The new PD-DTP—treatment and monitoring process.

Through PCN diagrams [48,49,50], the diagnosis process triggered by motor symptoms (Figure A6 in Appendix D), the prodromal diagnosis process (Figure A7 in Appendix D), the treatment and monitoring process of autonomous patients (patients who can self-administer the SensH&F test) (Figure A9 in Appendix D), and the treatment and monitoring process of not-autonomous patients (Figure A9 in Appendix D) were represented. In all PCN diagrams, the first and last process steps are colored gray.

To ensure a **Prodromal diagnosis** (WHY1), nurses at GP clinics would administer an olfactory test to over 60 patients every two years. The olfactory test can detect hyposmia (reduced olfactory sensitivity) in patients. Patients with hyposmia have an increased risk of developing PD [76,77]; thus, GPs would prescribe the SensH&F test to patients who test positive for the olfactory test. Nurses in territorial clinics would administer the SensH&F test. If the SensH&F test revealed suspicious values, the GP would refer the patient to a neurologist. The **Early diagnosis** (WHY2) process would be triggered by GPs prescribing SensH&F tests to people complaining of motor symptoms. The SensH&F test would be administered to patients with motor symptoms by nurses in territorial clinics. Regarding the **Periodic assessment** (WHY3) of patients’ motor performance, neurologists would prescribe SensH&F tests to patients at the end of each visit. Nurses in territorial clinics would administer SensH&F tests before the neurological visits to patients. Neurologists would be equipped with SensH&F to recheck motor performance if they deem it necessary. Finally, the **Frequent monitoring** of patients’ conditions (WHY4) appeared valuable in the presence of patients with motor fluctuations, patients undergoing the L-DOPA test, or patients whose therapy has been substantially changed [43]. SensH&F tests would be delegated, when possible, to patients with the help of their caregivers. In this case, a remote monitoring service would be activated. Neurologists would set up a testing session plan with SensH&F specific to each patient, and patients independently perform the testing meetings following the plan. If patients cannot self-administer the SensH&F test, nurses from territorial clinics would go to patients’ houses to administer it. Data acquired through SensH&F tests would be saved on the patient’s Electronic Health Record among all the PD-DTP, allowing the neurologist to update the testing session plan and/or change the therapy and/or schedule a visit.

Once the new PD-DTP was identified, it was tested in three meetings with stakeholders. In each session, a detailed presentation of the redesigned PD-DTP was provided, and stakeholders were asked to point out elements that they did and did not like, as well as elements that were unclear and/or could be improved [75]. The meetings revealed no critical problems.

**Table 6:** Motivation for main inclusion or exclusion decisions—WHY1 and WHY2.

Why	Decision	Motivation
*[WHY1]* *Prodromal diagnosis*	SensH&F tests are administered to patients over 60 with hyposmia (reduced olfactory sensitivity).	Testing all people older than 60 or familiar with PD would have been practically and economically unfeasible. Patients with hyposmia have an increased risk of developing PD [76,77]. SensH&F have already been proven to detect minor motor signs of PD in hyposmia patients [76,77]. Although patients with REM Sleep Behavior Disorder have an increased risk of developing PD, the complexity of diagnostic tools for this disorder does not make it suitable for screening [78,79].
	Olfactory tests are administered by nurses at GP clinics	Olfactory tests are cheap, fast, and easy to perform [80,81].
	Nurses in territorial clinics administer SensH&F tests.	Creation and consolidation of territorial facilities to oversee local communities’ health [82]. Patients in the prodromal stage are usually autonomous. Delegating SensH&F test administration to patients would involve providing SensH&F to and training a too vast number of patients. GPs are bottleneck resources. SensH&F test is simple neurophysiopatology technicians are a very scarce resource working solely within the hospital’s premises Paramedics are hardly qualified for the SensH&F test Nurses are qualified for the SensH&F test Assigning the SensH&F tests to nurses is consistent with the emergence of the *family health nurse* [82,83].
	GP is the care coordinator of this macro-stage: GP would prescribe an olfactory test to over 60 patients every two years. GP would prescribe the SensH&F test to patients who test positive for the olfactory test. GP would refer patients to a neurologist if the SensH&F test reveals suspicious values.	The GP is the only one with the opportunity and information to play this role.
*[WHY2]* *Early diagnosis*	GPs would prescribe the SensH&F test to people complaining of motor symptoms.	Patients typically go to the GP when they realize they have motor disorders.
	Nurses in territorial clinics would administer SensH&F tests for patients with motor symptoms.	All reasons given for prodromal diagnosis remain valid.

**Table 7:** Motivation for main inclusion or exclusion decisions—WHY3 and WHY4.

Why	Decision	Motivation
*[WHY3]* *Periodic assessment*	SensH&F test is decoupled from neurological examination: Neurologists would prescribe the SensH&F test to patients at the end of each visit. Nurses in territorial clinics would administer SensH&F tests before the neurological visits.	The decoupling would reduce the workload for the neurologist (a resource with very limited capacity) while allowing for more frequent monitoring of motor performance.
	Neurologists would be equipped with SensH&F if they want to double-check the motor performance.	Disagreement among neurologists on trusting the results of SensH&F test administered by others: “During the visit, the neurologist certainly does a part of the physical examination, but it would not make sense to repeat SensH&F test with SensH&F” (NEURO_1), “I would probably trust the measurement, but I would still do a motor performance check” (NEURO_3), “I would trust the measurement only if they were made by properly qualified people in accredited external agencies” (NEURO_7)), “SensH&F test and the neurological examination are complementary” (NEURO_8).
*[WHY4]* *Monitoring*	SensH&F tests are administered to patients with motor fluctuations, patients undergoing the L-DOPA test, or patients whose therapy has been substantially changed.	Having the opportunity to monitor motor symptoms of patients undergoing the L-DOPA test or patients whose therapy has been substantially changed would give neurologists essential information [43]
	Patients (with their caregivers) would self-administer SensH&F tests at home.	These patients may need to check their motor performance several times per day at different times of the day. Delegating SensH&F test administration to the physiotherapist is not viable since the interaction between the patient and the physiotherapist is usually insufficient.
	If patients cannot self-administer the SensH&F test, nurses from territorial clinics would go to patients’ houses to administer it.	The number of patients needing frequent monitoring and unable to self-administer SensH&F tests is small. *“In most cases, the patient and their informal caregiver are elderly, so traveling to territorial clinics to administer SensH&F tests would be a problem”* (ICR_2).
	Data acquired through SensH&F would be saved directly in the patient’s Electronic Health Record.	Data need to be easily accessible by neurologists Huge investments have strengthened Electronic Health Record usage [82,84].

## 4. Discussion

The new PD-DTP designed following the proposed five-phase framework addresses stakeholders’ needs and improves both the diagnosis and the treatment phases.

In terms of diagnosis, the improved PD-DTP will facilitate the earlier and more accurate identification of PD, potentially allowing for earlier interventions that may slow the disease’s progression. GPs will be better equipped to anticipate the diagnosis of PD and guide patients through the complex diagnostic journey. Neurologists will be able to diagnose PD earlier, more accurately, and with greater objectivity. Concerning the treatment, the motor status of patients will be assessed more frequently and precisely, enabling the assignment of tailored therapies. Neurologists will be able to monitor patients’ conditions more closely over time, allowing for precise adjustments in both pharmacological and non-pharmacological treatments, prioritizing patients based on changes in their health status. This refined, accurate, and objective assessment will benefit all T-Specialists, enhancing their ability to deliver patient-centered care. GPs will also be more informed in supporting patients through the PD-DTP process. Additionally, nurses will be empowered to provide improved care within their communities, aligning with recent national and European healthcare regulations [82,83]. Caregivers will also see indirect benefits through enhanced support and guidance, ultimately improving their quality of life. Lastly, the regional and national healthcare systems will optimize resource use and allocation (e.g., by reducing the burden on neurologists through the training of nurses, patients, and caregivers in the use of SensH&F) and contribute to overall higher quality of life for citizens.

Applying the framework to the case study has yielded positive outcomes for relevant stakeholders. MD developers have gained a deeper understanding of the potential service processes enabled by SensH&F, making them better equipped to promote and market the SensH&F to clinicians and healthcare institutions. Furthermore, the study provided valuable insights to MD developers on how SensH&F can be refined and enhanced. For instance, considering the potential use of SensH&F by patients at home, there is a clear need to integrate telemedicine systems and ensure the SensH&F are intuitive and accessible. Clinicians and healthcare managers involved in the study were able to thoroughly grasp the potential of the service processes enabled by SensH&F. This experience has increased their willingness to participate in future studies, such as clinical trials, and has even prompted some to consider purchasing SensH&F (once it is possible) for integration into their clinical practices, thereby accelerating technology adoption.

From a methodological point of view, applying the five-step framework to the case was successful. Indeed, the framework is more advantageous than the typical HCD and TED approaches when an MD has already been chosen. For example, starting the design process by identifying the key features of the MD allowed the design team to focus only on the needs that the chosen MD could meet without wasting stakeholders’ time discussing new service processes that would not be implemented. In addition, using the Problem–Benefit–Why matrix and the 5W coding scheme during Phase 4 allowed the design team to design new service processes that did not envision straightforward and easily predictable ways of employing the MD. The participatory nature of the framework enhances stakeholders’ involvement and, therefore, enables the identification of a solution that could benefit all stakeholders by leveraging the chosen MD and was approved by the stakeholders (Phase 5).

The flexible nature of the framework freed the design team to choose the tools that best fit the application. However, future users of the proposed approach must be cautious when selecting tools since the employed tools have a crucial role in the success of the design process. For example, the Power–Interest stakeholders’ matrix was chosen because PD-DTP is a long and complex service process, but it could be omitted in the case of simpler service processes. Similarly, PCN diagrams were employed to represent the current and new PD-DTP so that the interactions among the stakeholders in the service value network were explicit [49]. Still, other easier and more well-known visual frameworks (e.g., service blueprint [85] or patient journey [86]) are recommended in the case of less complex processes.

## 5. Conclusions

This work provides a framework for the HCD of service processes enabled by MDs. Such a methodology was applied to the design of a new PD-DTP enabled by SensH&F. Applying the five-phase framework to the case made it possible to test it successfully and to design a new PD-DTP that addresses stakeholders’ needs and improves all phases of the service process. This study has important theoretical and managerial implications. While there are several methodologies for incorporating stakeholders’ needs into MD development, the literature lacks methodologies that can support healthcare managers and MD developers in designing a service process enabled by new MDs, considering stakeholders’ perspectives. This study fills in this gap by presenting a framework that adds a TED perspective to the HCD one. Concerning the managerial implications, healthcare managers may be inspired by this methodology and seek to replicate it in their environment—across organizations, diseases, and MDs. Moreover, the framework can support MD developers who need to understand the impact of their MDs on the service processes they address, increasing the chances of MDs’ adoption in real settings. Finally, our results are directly relevant to administrators of PD service departments who want to improve their processes. Indeed, since the PD-DTP adopted in the Tuscany region (Italy) [62] is consistent with national [66] and international guidelines [61] for PD-DTP, this study provides a new PD-DTP that could inspire healthcare managers worldwide.

This paper is not without limitations. First, the proposed framework does not encompass the implementation and monitoring phases that are typically part of healthcare service process design [17,87,88]. While our framework focuses on the design phase, these additional phases could reveal valuable challenges and opportunities that might inform further refinements to the designed service process. Future research could provide a more comprehensive approach that includes these phases. For example, simulation or optimization tools could be added to show decision-makers the impact of the proposed solutions on the stakeholders involved before moving on to the implementation phase [89,90]. Second, only a general estimation of costs was obtained based on stakeholder opinions. Future studies could address this uncertainty and lack of data by applying a more rigorous evidence-based cost analysis [91,92]. Lastly, as the framework has been tested on a single case study, applying it to additional cases would help assess its generalizability and enable further refinements.

## Figures and Tables

**Table 2:** Case informants.

Informants	N	ID	Phases					Total Meetings
			1	2	4	5		
			Interview (I)	Focus Group (FG)	Brainstorming (BRAIN)	Co-Design (DES)	Test (TEST)	
Neurologists	8	NEURO_1	INT1	FG1		DES4		
		NEURO_2	INT2	FG1				
		NEURO_3	INT3	FG1	BRAIN4		TEST3	
		NEURO_4		FG2				
		NEURO_5		FG2				
		NEURO_6		FG2				
		NEURO_7				DES1		
		NEURO_8			BRAIN4			
PD patients	4	PAT_1	INT4					
		PAT_2				DES2		
		PAT_3				DES3		
		PAT_4					TEST1	
Informal caregivers	3	ICG_1	INT4					
		ICG_2				DES2		
		ICG_3				DES3		
Neurology department directors	1	NEUDIR_1				DES6		
Geriatricians	1	GER_1			BRAIN3			
Nuclear medicine specialists	1	NUCL_1			BRAIN3			
Neurosurgeons	1	NEUROSUR_1			BRAIN3			
General Practitioners	10	GP_1	INT5					
		GP_2	INT6					
		GP_3			BRAIN1			
		GP_4			BRAIN1			
		GP_5					TEST2	
		GP_6					TEST2	
		GP_7					TEST2	
		GP_8					TEST2	
		GP_9					TEST2	
		GP_10					TEST2	
Psychologists	3	PSY_1			BRAIN4			
		PSY_2			BRAIN4			
		PSY_3			BRAIN4			
Nurses	2	NU_1				DES6		
		NU_2				DES6		
Medical directors	1	MD_1				DES5		
Physiotherapists	1	PHYSIO_1			BRAIN4			
Territorial clinics managers	1	TCM_1			BRAIN4		TEST2	
Heads of the nursing staff	1	NURSEHEAD			BRAIN2			
Physiatrists	2	PHYSI_1			BRAIN4			
		PHYSI_2			BRAIN4			
Nurses with managerial duties	2	NURSEMNG_1			BRAIN2	DES6		
		NURSEMNG_2			BRAIN2			
Total informants	42							
Meetings per phase			6	2	4	6	3	21

**Table 3:** Problem–Feature–Benefit matrix.

Problem from Stakeholders’ Perspective (with Evidence from the Field)	Key Feature	Potential Benefit
[P1] Late diagnosis “*It was my son who had the concern because the same had happened to one of his friends*”—ICG_1. *“I couldn’t move my left foot properly. I thought it was caused by playing soccer. I went to the neurologist because my sister was diagnosed with PD 10 years ago”*—PAT_1. *“Initially, patients tend to hide problems, and the family calls me”—GP_2.*	[KF3] Fine-grained detection	[B1] Early detection
[P2] Subjective Assessment *“We use qualitative scales (mild, severe, or continuous) to measure tremor, which introduces significant inter- and intra-observer bias*”—GP_2.	[KF2] Objective detection	[B2] Objective assessment
[P3] Inaccurate Assessment *“The guidelines ask us to make judgments with the naked eye, which are, however, very inaccurate”—NEURO_2.*	[KF1] Accurate detection	[B3] Accurate assessment
[P4] Infrequent Assessment *“Patients are at risk of waiting three months or more to hear that therapy is not working*”—NEURO_2. *“The frequency with which patients are visited quite often does not depend on actual clinical needs but on the resources available*”—NEURO_3.	[KF4] Easy to use	[B4] Delegate
	[KF5] Easy to transport	[B5] Change setting
[P5] Improper Timing *“Motor symptoms usually worsen in the evening or early morning, but that’s not when we can visit the patient*”—NEURO_3.	[KF4] Easy to use	[B4] Delegate
	[KF5] Easy to transport	[B5] Change setting

**Table 4:** Need–Benefit matrix.

Stakeholders	Needs [N]	Potential Benefits				
		[B1] Early Detection	[B2] Objective Assessment	[B3] Accurate Assessment	[B4] Delegate	[B5] Change Setting
Persona 1—Giorgio (Main features: prodromal stage, no check-ups)	[N1] Be proactively involved in an early diagnosis process	X				
Persona 2—Elisabetta (Main features: initial motor symptoms, frequent check-ups)	[N2] Be subjected to the proper check-ups by the right physicians	X				
Persona 3—Simona (Main features: already diagnosed with PD, autonomous, with informal caregivers, capable of using technologies)	[N3] Continue doing the activities she loves		X	X	X	X
	[N4] Manage PD without reducing the quality of life of her family		X			
Persona 4—Paolo (Main features: already diagnosed with PD, low autonomy, with a formal caregiver, not capable of using technologies)	[N5] Strengthen the collaboration between doctors and caregivers		X			
	[N6] Receive therapies calibrated on the actual status of the disease		X	X	X	X
GPs	[N7] Activate a proactive diagnosis process	X				
	[N8] Effectively coordinate patient care		X	X	X	X
Neurologists	[N9] Obtain an early diagnosis	X				
	[N10] Avoid errors in the diagnosis and treatment process		X	X	X	X
	[N11] Facilitate patient’s handover to/from another neurologist		X	X		

**Table 5:** Problem–Benefit–Why matrix.

Problem	Potential Benefit	Why
[P1] Late diagnosis	[B1] Early detection	[WHY1] Prodromal diagnosis
		[WHY2] Early diagnosis
[P2] Subjective assessment	[B2] Objective assessment	[WHY3] Periodic assessment
[P3] Inaccurate assessment	[B3] Accurate assessment	[WHY3] Periodic assessment
[P4] Infrequent assessment	[B4] Delegate	[WHY4] Monitoring
	[B5] Change setting	[WHY4] Monitoring
[P5] Unproper timing	[B4] Delegate	[WHY4] Monitoring
	[B5] Change setting	[WHY4] Monitoring

**Figure A5:**
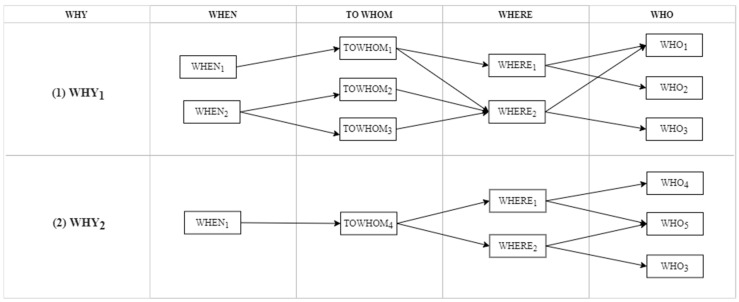
Generic example of the 5Ws coding schema.

**Figure A8:**
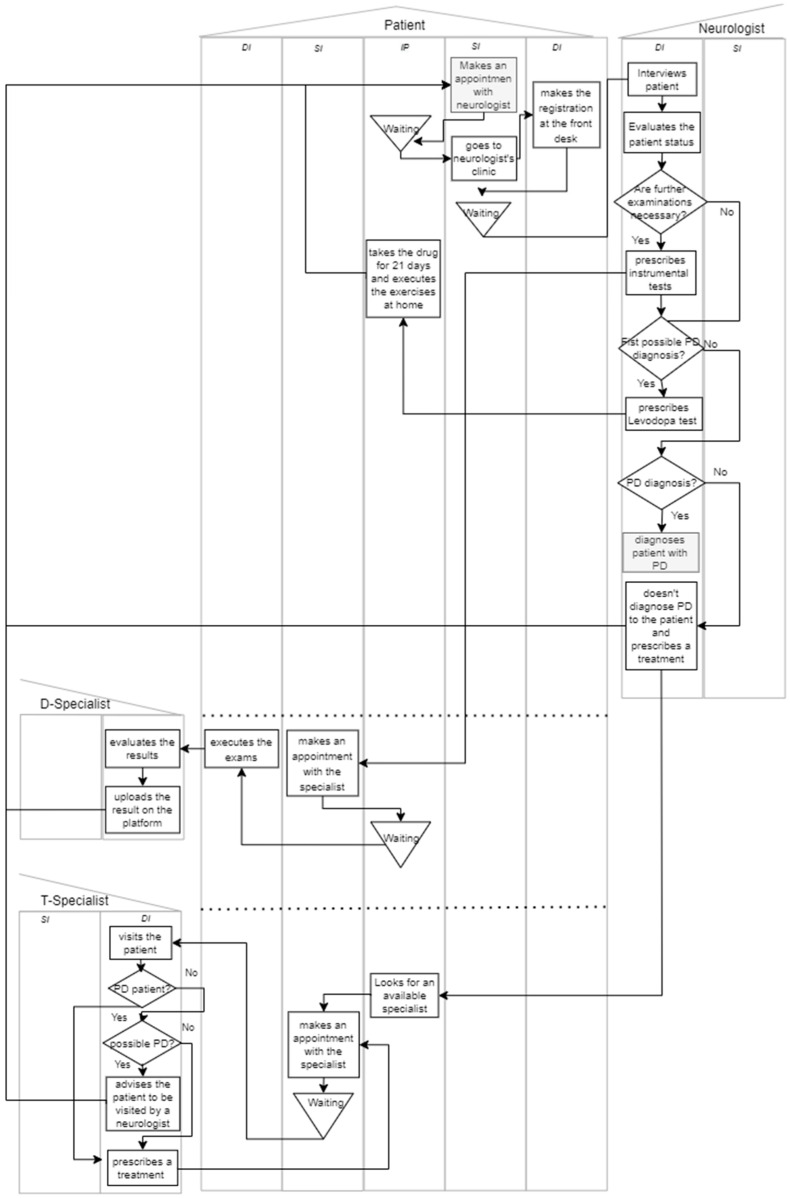
The new PD-DTP—neurological diagnosis.

## Data Availability

Additional materials concerning the meetings are available from the corresponding author upon reasonable request.

## Appendix C

Four purposely developed tools are suggested for carrying out some of the activities.

In Phase 2, the **Problem–Feature–Benefit matrix** (Table A1) is suggested to convert identified problems within the targeted service process into the potential benefits of the new service process. The conversion is made by considering the MD key features: thanks to the MD key feature, some of the identified problems could be addressed, and the new service process will offer potential benefits. The matrix is organized into three columns:

(1) Problem. This column lists the problems within the service process.
(2) Key Feature. This column identifies the MD key features that can mitigate or resolve the listed problems in the first column.
(3) Potential Benefits. This column outlines the anticipated benefits that can be realized when the MD’s key features address specific problems.

A single problem may require multiple MD key features for an effective solution. A single MD key feature may address multiple problems within the service process. The same potential benefit may arise from resolving different problems. There may be some problems that the MD cannot address, but they are not included in the matrix.

**Table A1:** Generic example of Problem–Feature–Benefit matrix.

Problem	Key Feature	Potential Benefit
P_1_	K_1_	B_1_
P_2_	K_k_	
…	…	…
Pp	K_1_	B_b_

In Phase 3, the **Need–Benefit matrix** (Table A2) is suggested to extract stakeholders’ needs that the identified potential benefits could address. A single need may require multiple potential benefits to be addressed. A single MD key feature may address multiple stakeholders’ needs. There may be some needs that the MD cannot address, but they are not included in the matrix. In the table, cells marked with ‘X’ indicate that a certain benefit B_x_ could address a certain need N_y_.

**Table A2:** Generic example of Need–Benefit matrix.

Stakeholders	Needs	Potential Benefits				
		B_1_	B_2_	B_3_	….	B_b_
S_1_	N_1_	X				
…	…					
Ss	N_n−1_		X	X		X
	N_n_		X			

In Phase 4, the **Problem–Benefit–Why matrix** (Table A3) is suggested to specify the role (“Why” column) the MD could have in addressing the identified problems through the potential benefits its use could offer.

**Table A3:** Generic example of Problem–Benefit–Why matrix.

Problem	Potential Benefit	Why
P_1_	B_1_	WHY_1_
		WHY_2s_
P_2_		WHY_2s_
…	…	…
P_p_	B_b_	WHY_w_

In Phase 4, the 5Ws coding schema (Figure A5) is suggested to collect and combine ideas for a new service process design. The framework involves codifying ideas according to five codes:

(1) Why (what could be the aim of the MD in the new service process?);
(2) When (when and how often may the MD be used?);
(3) To Whom (who may be the target population?);
(4) Where (in which setting may the MD be used?);
(5) Who (who may use the MD?).

Each row in Figure A5 corresponds to an aim (Why) an MD can have in the new service process. Each block corresponds to an idea about when (When), with which target population (to Whom), where (Where), and by whom (Who) the MD can be used to pursue the identified aim. Each “path” linking the different block types represents an alternative configuration for pursuing the identified aim. A new service process corresponds to a combination of one or more paths.
